# The Roles of Celiac Trunk Angle and Vertebral Origin in Median Arcuate Ligament Syndrome

**DOI:** 10.3390/diagnostics10020076

**Published:** 2020-01-31

**Authors:** Ryan P. Dyches, Kelsey J. Eaton, Heather F. Smith

**Affiliations:** 1 Department of Osteopathic Manipulative Medicine, Arizona College of Osteopathic Medicine, Midwestern University, Glendale, AZ 85308, USA; rdyches65@midwestern.edu (R.P.D.); keaton15@midwestern.edu (K.J.E.); 2 Department of Anatomy, Arizona College of Osteopathic Medicine, Midwestern University, Glendale, AZ 85308, USA; 3 School of Human Evolution and Social Change, Arizona State University, Tempe, AZ 85287, USA

**Keywords:** median arcuate ligament syndrome, celiac artery, celiac artery compression syndrome, diaphragm, superior mesenteric artery

## Abstract

Median arcuate ligament syndrome (MALS) is a rarely diagnosed condition resulting from compression of the celiac trunk (CT) by the median arcuate ligament (MAL) of the diaphragm. Ischemia due to reduced blood flow through the CT and/or neuropathic pain resulting from celiac ganglion compression may result in a range of gastrointestinal symptoms, including nausea, postprandial discomfort, and weight loss. However, the mechanism of compression and its anatomical correlates have been incompletely delineated. It has been hypothesized that CT angle of origination may be more acute in individuals with MALS. Here, frequency of anatomical variation in the MAL and CT were assessed in 35 cadaveric subjects (17M/18F), including the vertebral level of origin of CT and superior mesenteric artery (SMA), the distance between CT and MAL and SMA, the angles of origination of CT and SMA, the diameter at the CT base, and MAL/CT overlap. Females exhibited significantly higher rates of inferred MAL/CT overlap than males. Significant correlations were revealed between MAL/CT overlap and angles of origination of the CT and SMA. Vertebral level of origin of the CT in individuals with MAL/CT overlap was not significantly more superior than in those without. This study also revealed a significant relationship between MAL/CT overlap and angle of origination of the CT, which has clinical implications for understanding the anatomy associated with MALS.

## 1. Introduction

**Figure 1:**
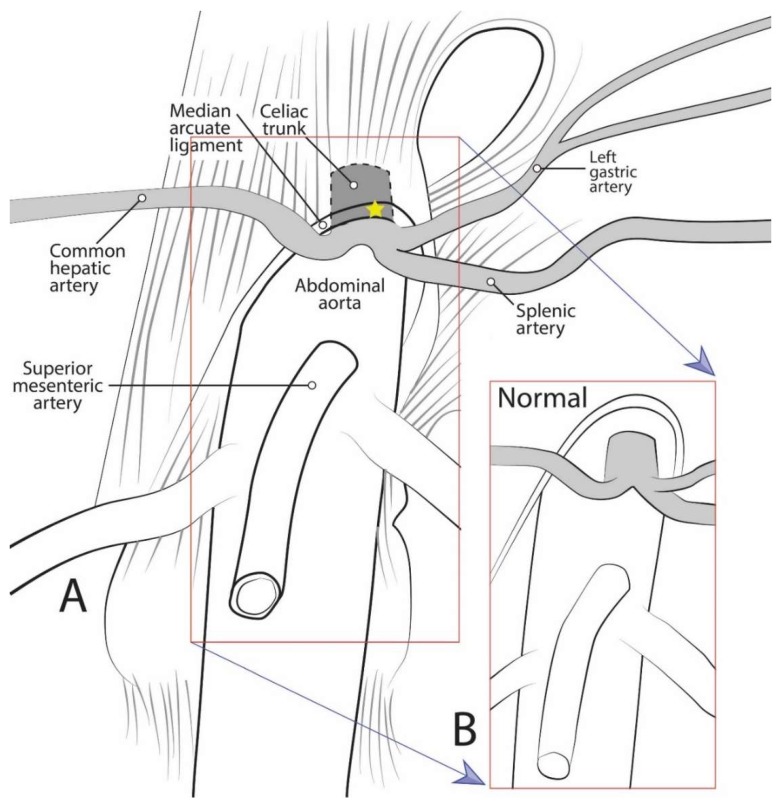
Diagrammatic representation of celiac trunk anatomy, showing: (**A**) The median arcuate ligament compressing the celiac trunk as in median arcuate ligament syndrome; and (**B**) normal anatomical condition.

Median arcuate ligament syndrome (MALS), also known as celiac artery compression syndrome or Dunbar syndrome, is a rarely diagnosed disease resulting from compression of the celiac trunk (CT) by the median arcuate ligament (MAL) [1] (Figure 1). The MAL is an arch-like fascial structure of the diaphragm linking the right and left diaphragmatic crura. While it has been noted as early as 1917 that the CT “is not infrequently partly covered by the diaphragm” [2], the precise relationship of the CT to the MAL was not extensively studied until recent years [3,4,5,6,7].

Embryologically, the abdominal diaphragm develops from its precursor, the septum transversum, which originates from mesenchyme in the mid-cervical region around embryonic day 22 [8]. Due to differential growth of the anterior and posterior regions of the embryo, the septum transversum appears to descend, and by week eight it reaches the level of the thoracic vertebrae [9]. The two crura (legs) of the diaphragm develop as muscular extensions that attach to the lateral sides of the lumbar vertebrae [9]. The crura are united in the midline by a tendinous band of fascia, the MAL. The aortic hiatus, an opening between the diaphragm and vertebral column around T12, permits passage of the abdominal aorta through the diaphragm [9]. Simultaneous to the development of the diaphragm, the CT, superior mesenteric artery (SMA), and inferior mesenteric artery (IMA) each form when the respective pair of developing segmental arteries converges at the midline of the abdominal aorta [8]. The CT develops to provide arterial supply to the foregut, the SMA to the midgut, and IMA to the hindgut. As the gut tube develops, the origins of these three unpaired visceral branches migrate caudally until they reach their final vertebral level around the end of month two [8]. The most common vertebral positions reported in adults are: T12 for the CT, L1 for SMA, and L3 for IMA [9]. Thus, the aortic hiatus, bounded by the MAL, frequently approximates the vertebral level of the CT, leaving little space between them. In some cases, the MAL may even overlap the CT, a condition which may result in impingement of the CT.

The reported incidence of MALS is low; however, it has been postulated that in 10% to 24% of the population, the MAL crosses the aorta at an atypically inferior anatomic level and subsequently results in compression by the MAL [10]. It is hypothesized that MALS-related pain is both neuropathic and due to vascular compression in cause; namely, foregut ischemia pain results from decreased blood flow and chronic compression, as well as overstimulation of the celiac ganglia contributing to sympathetic neuropathic pain [1]. The celiac ganglia are collateral sympathetic ganglia that lie adjacent to the CT along its anterolateral sides [9]. They are the largest sympathetic ganglia in the body, and provide the sympathetic innervation to the foregut organs [9]. While they are typically paired, there can be up to five celiac ganglia, all of which are connected via a complex neural network, the celiac plexus, receiving preganglionic sympathetic fibers from T5–T12 [9]. Since the celiac ganglia are responsible for sympathetic innervation to, and visceral pain from, the foregut, impingement of the ganglia can result in a range of symptoms including radiating foregut discomfort, nausea, vomiting, epigastric fullness, and delayed gastric emptying [1]. Thus, the MALS symptom complex may include a range of expressions resulting from both vascular ischemia and/or neuropathic pain and overstimulation.

The vast majority of patients with partial CT compression are asymptomatic because collateral circulation typically prevents the development of symptoms [11]. If the compression is severe and symptomatic, however, a diagnosis of MALS is considered. The typical MALS patient presentation is a young, thin woman with symptoms of nausea, vomiting, early satiety, difficulty gaining weight, postprandial epigastric pain, and an abdominal bruit [12,13]. Further workup with Doppler ultrasound and/or computed tomographic (CT-scan) imaging is required for definitive diagnosis [14,15,16,17]. Indication for surgery is established in these otherwise healthy, symptomatic patients by showing a baseline celiac velocity >200 cm/m^2^ and stenosis of the CA on CT-scan or MR angiography [12,13].

Although the superior mesenteric artery (SMA) is commonly described as originating 1.0 cm inferior to the CT, several studies have demonstrated the CT and SMA to be immediately adjacent in 22–58% of cases [3,4,18]. It is postulated that the close proximity of the two arteries may be evidence of CT compression by the MAL. In addition, one study found that up to 37% of cadavers had evidence of kinking of the CT [4]. A different study on fresh cadaveric specimens demonstrated that the CT origin was at or above the MAL in 33% of subjects [19]. However, it was unclear whether the CT originated more superiorly than normal, if the diaphragm extended further inferiorly, or if there was a combination of the two factors. A final possible indicator of CT compression is the CT angle of origination. It has been postulated that if there is an acute deviation of the left and right diaphragmatic crura, the MAL may cause constriction of the CT [20]. Despite these suggestions, the relationship between CT angle of origination and MAL compression has not been systematically assessed. This study aimed to increase the knowledge regarding the prevalence of MAL/CT overlap, the approximation and origin vertebral levels of the CT and the SMA, and the angle of origination of the CT, with the goal of better understanding the anatomical correlates of MALS. The study also sought to identify whether the vertebral level of the CT are associated with MAL/CT overlap.

## 2. Materials and Methods

### 2.1. Samples

Thirty-five cadaveric specimens (17M/18F) from the gross anatomy teaching laboratories at Midwestern University were assessed to determine the frequency of anatomical variation in the MAL and CT, especially MAL/CT overlap. Body donors ranged in age from 51–93 years old with a mean age of 77.4 years. Following student dissection, which included removal of the gastrointestinal organs, the integrity and presence of the MAL and CT were first evaluated. Additional dissection and preparation were necessary to further reveal the MAL and its associated neurovascular structures in several cadavers. Cadavers were embalmed with 10% formaldehyde and standard embalming protocols. Any specimens with gross gastrointestinal (GIT) abnormalities were removed from consideration. This study was determined to be IRB-exempt by the Midwestern University Institutional Review Board, due to the subjects being entirely cadaveric (#AZ 1259).

### 2.2. Data Collection

Data were then collected on several variables relating to size and relative positions of the structures of interest. First, the vertebral levels of the CT and SMA were assessed via manual palpation of the ribs and vertebrae. The CT was assessed for any evidence of overlap of the CT by the MAL or diaphragmatic crura by measuring the distance between the two structures. Negative distances were interpreted to indicate overlap. The diameter of the celiac trunk at its origin from the aorta and the distances between the CT and each of the MAL and SMA were measured using Mitutoyo sliding digital calipers. Finally, the angles of origination of the CT and SMA were measured using the U Protractor application on an iPhone.

### 2.3. Statistical Analyses

A series of statistical analyses were conducted to evaluate MAL/CT overlap and the other variables, and to determine whether sex differences existed in each variable. In particular, Chi-squared analyses were conducted to determine whether significant differences in frequency of MAL/CT overlap existed between the sexes. Analyses of Variances (ANOVA) were conducted to assess whether sex differences existed in any of the other variables, and to determine whether individuals with MAL/CT overlap exhibited significant differences in any of the other variables. Correlation analyses were performed to assess the relationship among each pair of variables. Since it is possible that some anatomical variables change with age, Partial Correlation analyses were also conducted controlling for age. All statistical analyses were performed using SPSS 25 (IBM Corp, Armonk, NY USA).

## 3. Results

### 3.1. Comparison of Sexes and Rates of MAL/CT Overlap

**Figure 2:**
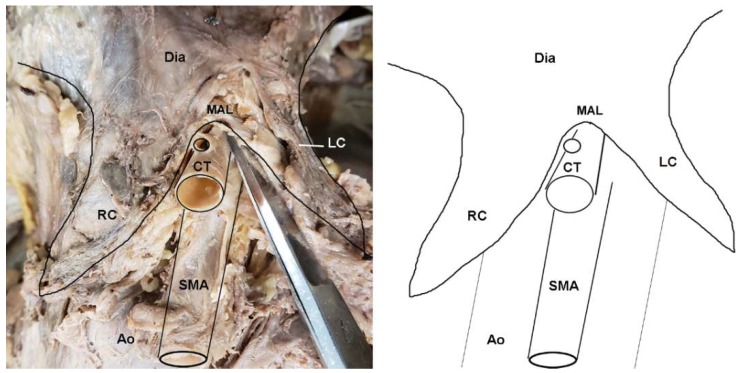
Dissection photo showing compression of the celiac trunk by the median arcuate ligament in a cadaveric subject: (**left**) Photo; (**right**) illustration. Abbreviations are as follows: Ao = aorta; CT = celiac trunk; Dia = diaphragm; LC = left crus of diaphragm; MAL = median arcuate ligament; RC = right crus of diaphragm; SMA = superior mesenteric artery.

Approximately one-third of subjects (31.4%) were found to exhibit a morphology in which the diaphragm extended further inferiorly than the base of the CT, at least partially covering it (Figure 2; Table S1). Chi-squared tests revealed a highly significant difference in MAL/CT overlap between the sexes (Table 1; X2 = 12.39; *p* < 0.001), with females exhibiting a significantly higher frequency of MAL/CT overlap than males (44.4% vs. 21.4%). An ANOVA revealed significant differences in the origination angles of both CT (F = 7.256, *p* = 0.011) and SMA (F = 15.084, *p* < 0.001) between individuals with MAL/CT overlap and those without. However, there was no significant difference in the vertebral level of the CT or SMA between individuals with MAL/CT overlap versus those without. There was also no significant difference in diameter of CT or in the distance between the CT and SMA.

### 3.2. Regression Results

**Figure 3:**
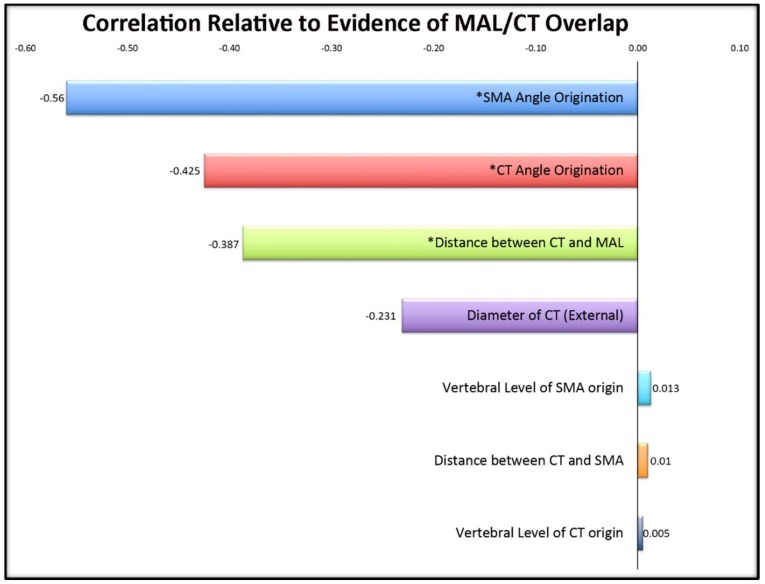
Correlations of cadaveric variables in relation to evidence of MAL/CT overlap. Significant values (*p* < 0.05) are denoted with an asterisk (*). Its significant negative correlation indicates that a smaller angle of origination suggests a greater likelihood of MAL/CT overlap.

The regression analyses revealed correlations between age and: external diameter of the celiac trunk (*r* = 0.500, *p* = 0.002), and angle of origination of the CT (*r* = 0.467, *p* = 0.005). Therefore, we also conducted a second set of analyses controlling for age through a partial correlation. When age was factored out, significant correlations were revealed between MAL/CT overlap and angles of origination of both the CT (*r* = 0.466, *p* = 0.005) and SMA (*r* = 0.439, *p* = 0.009), further supporting the results from the ANOVA (Table 2; Figure 3).

### 3.3. Clinically Relevant Variants Identified

**Figure 4:**
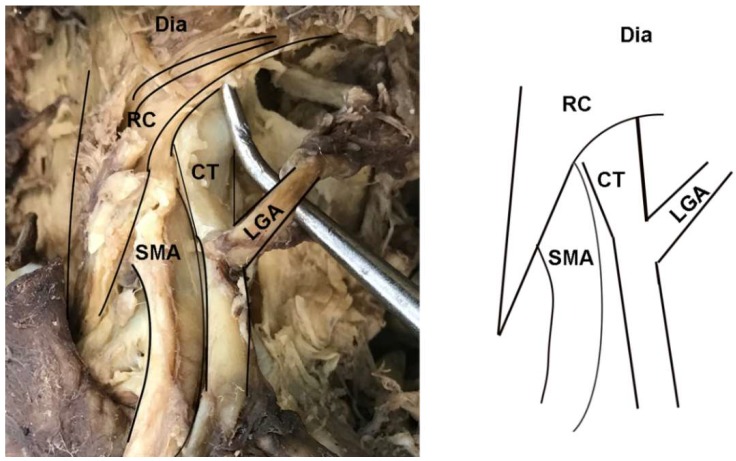
Dissection photo showing overlap of the celiac trunk by the right crus of the diaphragm: (**left**) Photo; (**right**) illustration. Abbreviations are as follows: CT = celiac trunk; Dia = diaphragm; LGA = left gastric artery; RC = right crus of diaphragm; SMA = superior mesenteric artery.

**Figure 5:**
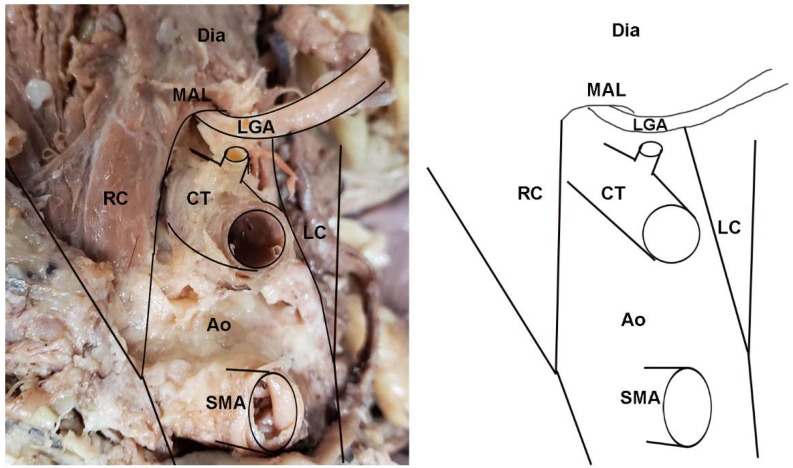
A notable variant displaying an uncommon branching of the left gastric artery (LGA) directly off the aorta in a cadaveric specimen: (**left**) Photo; (**right**) illustration. This example also demonstrates the direct overlap of the LGA by the median arcuate ligament. Abbreviations are as follows: Ao = aorta; CT = celiac trunk; Dia = diaphragm; LC = left crus of diaphragm; LGA = Left gastric artery; MAL = median arcuate ligament; RC = right crus of diaphragm; SMA = superior mesenteric artery.

In addition to instances of MAL/CT overlap, several other clinically relevant anatomical variants of the CT were observed in the sample. In two female specimens, the CT arose deep to one of the diaphragmatic crura and coursed through its fibers to emerge into the abdominal cavity (Figure 4). These cases were tallied as instances of MAL/CT overlap; however, the mechanism of overlap was the crus rather than MAL. One male specimen displayed a rare condition of the left gastric artery branching directly off the abdominal aorta, rather than off the CT (Figure 5). In this case, the left gastric artery arose approximately 1 cm superiorly to the CT, and was covered by the MAL.

## 4. Discussion

### 4.1. Angle of Celiac Trunk

The significant findings of this study, seen in Figure 3, demonstrate that when the CT angle of origin was more acute, there was an increased occurrence with MAL/CT overlap. Importantly, we believe this angle is the demonstration of the overlap, not the cause, in light of the correlation with the distance from the MAL implying the close proximity of the ligament. This confirms the source of overlap as being structurally induced by the ligament rather than in relation to an inherent angle.

### 4.2. Vertebral Origin of Celiac Trunk

Other studies have explored the branching height of the CT as it bifurcates from the abdominal aorta. It intuitively makes sense that with a more proximal branching point, the trunk and corresponding arteries will need to travel more acutely to exit from underneath the MAL. However, our findings did not support this hypothesis. As seen in Table 2, no correlation was found between the vertebral height of the CT and the observed MAL/CT overlap. This finding suggests that the mechanism of overlap may be the morphology of the MAL and diaphragm, rather than the height of the CT. In other words, individuals with MALS are more likely to have an inferiorly positioned or elongated diaphragm than a high CT. This interpretation is further supported by the two examples in which the CT was otherwise normally-positioned, but still emerged through the left crus of the diaphragm. Future studies comparing length of the crura between individuals with and without MAL/CT overlap could clarify this relationship.

### 4.3. Clinical Implications of MALS Findings

MALS significantly affects multiple health aspects of afflicted patients, creating both physical and emotional detriment. Patients undergo not only severe pain but the frustrations of symptomatology affecting activity participation, social interactions, as well as an effect on multiple body systems due to nutritional deficit. The diagnosis continues to be an elusive topic of understanding. Various established researchers and journals have ventured into determining its prevalence and clinical significance, yet its legitimacy is still debated. Previous literature has demonstrated high rates of variable CT compression by the MAL, in 10–50% of cases [19,21]. True symptomatology is minimal and intervention success remains imperfect, with only 50–80% of symptoms resolving after surgical release [22]. The deliberation over the validity of this syndrome lies within whether there is an undefined comorbid condition, as not all patients with compression visible on CT demonstrate symptoms [16]. Our results suggest that the angle of origination of the CT from the aorta may be useful as part of the diagnostic assessment of possible cases of MALS. This angle can be reliably determined using contrast-enhanced computed tomographic angiography (CTA). The goal is that with improved specificity of diagnostic measures, patients’ conditions may be more rapidly treated and with greater confidence when using invasive measures.

Per prior reports, symptomatology is an important correlation when assessing compression. Similar to the phenomenon of magnetic resonance imaging (MRI) exposing vertebral disc abnormalities in >50% of asymptomatic individuals 30–39 years of age with disc degeneration [23], the MAL may apply compression without symptomology. An additional difficulty arises in that not all subjects express symptoms in a consistent manner. In a study by Harr et al. in 2014, young athletes were more likely to describe a stich feeling rather than the “classic” nausea, vomiting, and abdominal pain [24]. This subset of patients may already display a thin body habitus due to their athletic activities which may confound the classic presentation of MALS. Care providers should be aware of MALS in thin female athletes whose performance suddenly drops without otherwise evident causes.

### 4.4. Clinical Implications of Additional Anatomical Variants Identified

In addition to instances of full CT overlap by MAL, this study also revealed an alternate example of overlap of the foregut arterial supply by the diaphragm. In one specimen, the left gastric artery arose from the aorta superior to the CT and was covered by the MAL. While this condition would likely not be associated with the full range of symptoms as overlap of the entire CT, it could nonetheless result in reduced blood supply to the stomach, especially along the lesser curvature. This ischemia could result in similar, albeit less severe, symptoms of MALS, such as early satiety, postprandial discomfort, and/or nausea. However, in this condition, the celiac ganglion would likely be spared from overstimulation, and the blood flow to the liver, spleen, and pancreas would not be compromised.

### 4.5. Limitations

Our use of cadaveric specimens allows for assessment of arterial angles without diaphragmatic motion effect or muscular tension. While this is useful in assessing for pure angulation of the branching point, we recognize that this limits the assessment of the effects of breathing patterns on arterial compression, one that is predominantly found in exhalation. While a limitation, it is important to note that the ligament is the least mobile part of the diaphragm. Similarly, it is conceivable that dissecting these structures could alter their natural position in the body, or that the act of embalming the cadaver could alter the arterial angles. Further study of in vivo subjects, CT angiogram, and correlation with symptomatology is the next progression in this study, and will further elucidate whether CT angle is directly correlated with symptomatology. Finally, it should be acknowledged that the sample size is not large and is necessarily skewed in the direction of older aged individuals. However, within the sample, a broad range of stature, weight, and morphology was observed, suggesting that the sample captures as reasonably representative sample of older Americans.

## 5. Conclusions

This study revealed a high degree of variability in the relationship between the MAL and CT, identifying a high frequency of MAL/CT overlap. It also confirmed that MAL/CT overlap occurs significantly more often in women. While this study did not specifically address the reason for this sex difference, it may be related to an overall lower percentage of abdominal fat in women. Additionally, it revealed that MAL/CT overlap is not generally associated with a superiorly positioned CT. The angle correlation, a new perspective, allows another means of assessment in conjunction with the preexisting criteria of post stenotic dilation, flow variance, constriction. It is our belief that this information will allow for increased specificity in future studies of those with symptomatology, so as to aid in intervention decisions.

## Figures and Tables

**Table 1:** Cadaveric data on anatomical variables measured broken down by sex and MAL/CT overlap status.

	Origin CT	Origin SMA	Distance CT-MAL (mm)	Distance CT-SMA (mm)	Diameter CT (mm)	Angle Origination CT	Angle Origination SMA
Females	**T11 = 16.7%, T12 = 50.0%, L1 = 33.3%**	**T12 = 38.9%, L1 = 38.9%,** **L2 = 22.2%**	4.6	11.2	8.0	58.6°	58.4°
Males	**T11 = 23.5%, T12 = 52.9%, L1 = 23.5%**	**T12 = 47.1%, L1 = 47.1%,** **L2 = 5.9%**	8.7	10.0	8.9	72.1°	59.1°
MAL/CT overlap	**T11 = 18.2%, T12 = 54.5%, L1 = 27.3%**	**T12 = 54.5%, L1 = 18.2%,** **L2 = 27.3%**	11.1	10.6	8.7	72.4°	67.4°
MAL/CT non-overlap	**T11 = 20.8%, T12 = 50.0, L1 = 29.2%**	**T12 = 37.5%, L1 = 54.2%,** **L2 = 8.3%**	−3.3	10.7	7.8	49.4°	39.9°
**Mean**	**T11= 20%, T12= 51.4%, L1 = 28.6%**	**T12 = 42.9%, L1 = 14.3%,** **L2 = 42.9%**	**6.6**	**10.6**	**8.4**	**65.1°**	**58.8**

Abbreviations: CT = celiac trunk; MAL = median arcuate ligament; SMA = superior mesenteric artery. Mean values indicated in bold text.

**Table 2:** Results of partial correlation analyses controlling for age among variables. Significant values are indicated in bold. It is interesting to note that the vertebral origin of the CT and SMA did not yield significant values.

	Diameter CT	Angle Origination CT	Angle Origination SMA	MAL/CT Overlap
Sex	0.247 (*p* = 0.152)	0.269 (*p* = 0.118)	0.15 (*p* = 0.933)	−0.289 (*p* = 0.093)
Vertebral origin CT	−0.022 (*p* = 0.900)	0.126 (*p* = 0.472)	−0.059 (*p* = 0.738)	0.005 (*p* = 0.977)
Vertebral origin SMA	−0.198 (*p* = 0.254)	−0.069 (*p* = 0.693)	0.082 (*p* = 0.640)	0.013 (*p* = 0.943)
Distance CT-MAL	**0.334 (*p* = 0.050)**	**0.360 (*p* = 0.033)**	0.170 (*p* = 0.329)	**−0.387 (*p* = 0.022)**
Distance CT-SMA	**−0.385 (*p* = 0.022)**	−0.201 (*p* = 0.247)	0.277 (*p* = 0.107)	0.010 (*p* = 0.956)
Diameter CT		**0.467 (*p* = 0.005)**	−0.009 (*p* = 0.960)	−0.231 (*p* = 0.182)
Angle origination CT			0.313 (*p* = 0.067)	**−0.425 (*p* = 0.011)**
Angle origination SMA				**−0.560 (*p* < 0.001)**

Abbreviations: CT = celiac trunk; MAL = median arcuate ligament; SMA = superior mesenteric artery.

## Supplementary Materials

The following are available online at https://www.mdpi.com/2075-4418/10/2/76/s1, Table S1: data file.
